# Treatment Preferences for Internet-Based Cognitive Behavioral Therapy for Insomnia in Japan: Online Survey

**DOI:** 10.2196/12635

**Published:** 2019-05-15

**Authors:** Daisuke Sato, Chihiro Sutoh, Yoichi Seki, Eiichi Nagai, Eiji Shimizu

**Affiliations:** 1 Department of Cognitive Behavioral Physiology Graduate School of Medicine Chiba University Chiba Japan; 2 Department of Rehabilitation Sciences Chiba Prefectural University of Health Sciences Chiba Japan; 3 Cognitive Behavioral Therapy Center Chiba University Hospital Chiba Japan; 4 Clinical Research Center Chiba University Hospital Chiba Japan; 5 Research Center for Child Mental Development Chiba University Chiba Japan

**Keywords:** patient preference, insomnia, internet-based cognitive behavioral therapy

## Abstract

**Background:**

The internet has the potential to increase individuals’ access to cognitive behavioral therapy (CBT) for insomnia at low cost. However, treatment preferences regarding internet-based computerized CBT for insomnia have not been fully examined.

**Objective:**

The aim was to conduct an anonymous online survey to evaluate treatment preferences for insomnia among patients with insomnia and individuals without insomnia.

**Methods:**

We developed an online survey to recruit a total of 600 participants living in the Kanto district in Japan. There were three subgroups: 200 medicated individuals with insomnia, 200 unmedicated individuals with insomnia, and 200 individuals without insomnia. The survey asked questions about the severity of the respondent’s insomnia (using the Athens Insomnia Scale), the frequency of sleep medication use and the level of satisfaction with sleep medication use, the respondent’s knowledge of CBT, his or her preference for CBT for insomnia before drug therapy, preference for CBT versus drug therapy, and preference for internet-based CBT versus face-to-face CBT.

**Results:**

Of the 600 respondents, 47.7% (286/600) indicated that they received CBT before drug therapy, and 57.2% (343/600) preferred CBT for insomnia to drug therapy. In addition, 47.0% (282/600) preferred internet-based CBT for insomnia to face-to-face CBT. Although the respondents with insomnia who were taking an insomnia medication had a relatively lower preference for internet-based CBT (40.5%, 81/200), the respondents with insomnia who were not taking an insomnia medication had a relatively higher preference for internet-based CBT (55.5%, 111/200).

**Conclusions:**

The results of our online survey suggest that approximately half of the people queried preferred CBT for insomnia to drug therapy, and half of the respondents preferred internet-based CBT for insomnia to face-to-face CBT.

## Introduction

According to the *Diagnostic and Statistical Manual of Mental Disorders* (Fifth Edition), insomnia is a sleep disorder characterized by recurrent poor sleep quality or quantity that causes distress or impairment in important areas of functioning. Epidemiological studies suggest that the prevalence of clinical insomnia disorder ranges from 10% to 12% [1, 2], and the problems are often long-lasting [1]. There are two treatment options for individuals who have insomnia: cognitive behavioral therapy (CBT) [2-4] and pharmacotherapy, including nonbenzodiazepines, benzodiazepines, melatonin agonists, and an orexin receptor antagonist. In their systematic review, Mitchell et al [5] stated that over the long term the effectiveness of CBT for insomnia is superior to the effectiveness of benzodiazepine and non-benzodiazepine drugs. It is worthwhile to note that benzodiazepines are more frequently prescribed in Japan than in any other country [6].

Although there is evidence that pharmacological treatments improve insomnia (especially in the short term), these treatments have some significant potential adverse effects, including residual sedation and memory impairment. Patients with insomnia often report a preference for CBT. For example, Vincent and Lionberg [7] reported that at pretreatment in an outpatient hospital setting in Canada, CBT was significantly preferred over pharmacological therapy by 43 participants based on overall acceptability ratings. In a study from Australia, Walters et al [8] showed that a series of individuals with schizophrenia or schizoaffective disorders preferred CBT when given the choice of pharmacotherapy, melatonin, and CBT.

A problem related to CBT is that access to face-to-face CBT is extremely limited in some regions, including Japan, due to human resource and expertise constraints [9-12]. Accumulating evidence in recent years suggests that internet-based, computerized CBT for insomnia can be an effective treatment [13-15], and attention has turned to internet-based CBT for insomnia as an alternative to face-to-face CBT [16]. Web programs are accessible independent of the user’s location and can be conducted on one’s own time and at a low cost. Although the potential preference for CBT for insomnia over pharmacotherapy has been investigated in Western countries, there have been no studies of treatment preferences among individuals with and without insomnia in Japan [17,18]. In this study, we conducted an anonymous online survey to evaluate treatment preferences for insomnia among medicated individuals with insomnia, nonmedicated individuals with insomnia, and individuals without insomnia.

## Methods

### Survey Respondents

We had an online research agency (Cross Marketing Inc, Tokyo) oversee our Web-based survey. After being provided a thorough understanding of our research and agreeing to voluntarily participate in the study, 600 participants were recruited from the Kanto district in Japan through the online research provider. They consisted of 200 individuals with insomnia who were using a medication for insomnia, 200 individuals with insomnia who were not taking any insomnia medication, and 200 individuals without insomnia by their self-reports. Each group of 200 participants consisted of 100 men, including 20 men each in their twenties, thirties, forties, fifties, and sixties, and 100 women, including 20 women each in their twenties, thirties, forties, fifties, and sixties, to match the groups by age and gender.

### Procedure

The candidate respondent received brief text-based information about the survey, including its objectives and conditions for participation (informed consent). The survey consisted of two parts. The first part asked demographic questions including gender, age, living area, employment status, and presence and severity of insomnia using the Athens Insomnia Scale (AIS), a self-reported psychometric questionnaire consisting of eight items developed by Soldatos et al [19].

The survey’s second part consisted of the following questions about treatment for insomnia.

Frequency of sleep medication use and the level of satisfaction with sleep medication use:

1. How often do you take medication for insomnia?
2. Are you satisfied with your current insomnia medication?

Knowledge of CBT:

1. Have you heard of cognitive behavioral therapy (CBT)? Did you know that CBT is also a treatment for mental disorders such as depressive disorders and anxiety disorders?
2. As a treatment for insomnia, it has been shown that cognitive behavioral therapy (CBT) is medically effective. Did you know that CBT is effective for insomnia?

Preference for CBT before drug therapy, preference for CBT versus drug therapy, and preference for internet-based CBT versus face-to-face CBT:

1. Let’s assume that you received your diagnosis of insomnia from your doctor. Imagine that you were advised to take cognitive behavioral therapy (CBT) before drug therapy. In such a case, have you received CBT?
2. If you had to choose either CBT for insomnia or drug therapy for insomnia, which would you choose?
3. Aside from the method of CBT for insomnia in face-to-face sessions with a therapist, if there is a way to receive CBT for insomnia via a computer program on the internet and support from a therapist by email, which would you choose?

### Statistical Analysis

We used descriptive analyses (numbers, frequencies, percentages, means and standard deviations). We compared differences in each survey item among the three respondent groups, using the chi-square test, ANOVA, and a residual analysis [20]. An alpha level of .05 was used. All data were analyzed using SPSS for Windows version 21 (SPSS, Chicago, IL, USA).

### Ethical Approval

The study was approved by the Regional Ethical Review Board, Faculty of Medicine, Chiba University (2017-5-19; No. 2711).

## Results

### Demographic Characteristics of the Survey Respondents

As designed, a total of 600 respondents (300 men and 300 women; mean age 45, SD 14 years, range 20-69 years) completed the online survey (Table 1). As shown by the respondents’ use of the AIS, the respondents with insomnia who were taking an insomnia medication and respondents with insomnia who were not taking an insomnia medication had significantly more severe insomnia compared to the respondents without insomnia. When we defined an AIS total score of 6 or higher as insomnia, 90.0% (180/200) of the respondents with insomnia were using an insomnia medication and 94.5% (189/200) of the respondents with insomnia were not taking an insomnia medication compared to 52.0% (104/200) of the respondents without insomnia (see Table 1). There were also significant differences among the three respondent groups in employment status and AIS score (see Table 1).

**Table 1:** The demographic characteristics of the 600 survey respondents.

Variable		With insomnia, using medication (n=200)	With insomnia, not using medication (n=200)	Without insomnia (n=200)	χ^2^ (df)	*F* value (df1,df2)	*P* value
**Sex, n (%)**					**0.0 (2)**		**>.99**
	Women	100 (50.0)	100 (50.0)	100 (50.0)			
	Men	100 (50.0)	100 (50.0)	100 (50.0)			
Age (years), mean (SD)		45 (14)	45 (14)	45 (14)		0.01 (2,398)	>.99
**Employment status**					**0.0 (10)**		**<.001**
	Full-time	66 (25.9)	98 (38.4)	91 (35.7)			
	Part-time	34 (41.0)	19 (22.9)	30 (36.1)			
	Self-employed	11 (55.0)	2 (10.0)	7 (35.0)			
	Housewife	25 (28.7)	36 (41.4)	26 (29.9)			
	Unemployed	43 (45.3)	30 (31.6)	22 (23.2)			
	Other	21 (35.0)	15 (25.0)	24 (40.0)			
AIS^a^ score, mean (SD)		10.31 (4.46)	10.28 (3.60)	5.96 (3.14)		87.97 (2,597)	<.001
Insomnia (AIS ≥6)		180 (38.1)	189 (40.0)	104 (22.0)	130.7 (2)		<.001

^a^AIS: Athens Insomnia Scale.

### Level of Satisfaction With Sleep Medication Use

The respondents with insomnia who were taking an insomnia medication (n=200) were asked about the frequency of their insomnia medication use; 68.0% (136/200) reported that they used an insomnia medication every night, and 95.5% (191/200) reported using such a medication at least once per week (Table 2). The respondents who indicated that they used an insomnia medication were also asked about their level of satisfaction with the sleep medication use: 54.0% (108/200) were satisfied, 27.5% (55/200) were neutral, and 18.5% (37/200) were dissatisfied.

**Table 2:** Frequency of sleep medication use and level of satisfaction with sleep medication use among the 200 respondents with insomnia who were using an insomnia medication.

Questions and answers		Respondents, n (%)
**How often do you take medicine?**		
	Every night (time per day)	136 (68.0)
	3-4 times per week	40 (20.0)
	1 time per week	15 (7.5)
	1 time per 2 weeks	4 (2.0)
	1 time per month	1 (0.5)
	<1 time per 2 months	4 (2.0)
**Are you satisfied with your current medicine?**		
	Very satisfied	35 (17.5)
	Satisfied	73 (36.5)
	Neutral	55 (27.5)
	Dissatisfied	28 (14.0)
	Very dissatisfied	9 (4.5)

### Knowledge of Cognitive Behavioral Therapy

All respondents were asked about their knowledge of CBT. There were significant differences among the three respondent groups in their knowledge of CBT and the effects of CBT (Table 3): 55.0% (330/600) of the respondents had no knowledge of CBT. Among the three groups, the percentage of those who had no knowledge of CBT were as follows: 39.0% (78/200) of the respondents with insomnia who were taking an insomnia medication, 54.0% (108/200) of the respondents with insomnia who were not taking an insomnia medication, and 72.0% (144/200) of the respondents without insomnia. In the group of respondents with insomnia who were using medication, the response “I have heard of CBT, and I know that it is an insomnia treatment” was significantly more frequent comparing the three respondent groups’ answers. Conversely, the response “I have never heard of CBT, and I did not know that it is an insomnia treatment” was significantly less comparing the three respondent groups’ answers. Among the group of respondents with insomnia who were not taking insomnia medication, the response “I have heard of CBT, and I know that it is an insomnia treatment” was significantly less frequent comparing the three respondent groups’ answers, and “I have heard of CBT, and I did not know that it is an insomnia treatment” was significantly more frequent comparing the three respondent groups’ answers. In the group without insomnia, the response “I have heard of CBT, and I know that it is an insomnia treatment” was significantly more frequent, and the response “I have never heard of CBT, and I did not know that it is an insomnia treatment” was significantly less frequent comparing the three respondent groups’ answers (Table 4).

Even among the respondents who had heard of CBT (n=270), 68.5% did not know that CBT is effective for insomnia. Among the respondents with insomnia who were using an insomnia medication, the response “Do you know that CBT is effective for insomnia? Yes, I know” was significantly more frequent comparing the three respondent groups’ answers, and “No, I do not know” was significantly less frequent comparing the three respondent groups’ answers. Among the survey respondents with insomnia who were not using an insomnia medication, the response “Do you know that CBT is effective for insomnia? Yes, I know” was significantly less frequent comparing the three respondent groups’ answers, and “No, I do not know” was significantly more frequent comparing the three respondent groups’ answers (Table 3).

**Table 3:** Knowledge of cognitive behavioral therapy (CBT) in general.

Question and answers		With insomnia, using medication (n=200)		With insomnia, not using medication (n=200)		Without insomnia (n=200)		Total (N=600)	
		n (%)	Adjusted residual	n (%)	Adjusted residual	n (%)	Adjusted residual	n (%)	*P* value^a^
**Have you heard of cognitive behavioral therapy (CBT)? Did you know that it is a treatment for mental disorders such as depression disorders and anxiety disorders?**									
	I have heard of CBT, and I know that it is an insomnia treatment	82 (41.0)	7.9^b,c^	32 (16.0)	−2.5^b,d^	18 (9.0)	−5.4^b,c^	132 (22.0)	<.001
	I have heard of CBT, but I did not know that it is an insomnia treatment	40 (20.0)	−1.2	60 (30.0)	2.9^b,c^	38 (19.0)	−1.6	138 (23.0)	
	I have never heard of CBT, and I did not know that it is an insomnia treatment	78 (39.0)	−5.6^b,c^	108 (54.0)	−0.3	144 (72.0)	5.9^b,c^	330 (55.0)	

^a^From Pearson chi-square values.

^b^Cells with significant adjusted standardized residuals.

^c^The adjusted standardized residual is 2.58 or greater (or, alternatively, less than −2.58), its associated probability is less than 0.01.

^d^The adjusted standardized residual is 1.96 or greater (or, alternatively, less than −1.96), its associated probability is less than 0.05.

**Table 4:** Knowledge of cognitive behavioral therapy (CBT) for insomnia.

Question and answers		With insomnia, using medication (n=122)		With insomnia, not using medication (n=92)		Without insomnia (n=56)		Total (N=270)	
		n (%)	Adjusted residual	n (%)	Adjusted residual	n (%)	Adjusted residual	n (%)	*P* value^a^
**As a treatment for insomnia, it has been shown that CBT is medically effective. Did you know that CBT is effective for insomnia?**									
	Total	122 (100.0)		92 (100.0)		56 (100.0)		270 (100.0)	<.001
	Yes, I know	54 (44.3)	4.1^b,c^	18 (19.6)	−3.0^b,c^	13 (31.5)	−1.5	85 (31.5)	
	No, I did not know	68 (55.7)	−4.1^b,c^	74 (80.4)	3.0^b,c^	43 (68.5)	1.5	185 (68.5)	

^a^From Pearson chi-square values.

^b^Cells with significant adjusted standardized residuals.

^c^The adjusted standardized residual is 2.58 or greater (or, alternatively, less than −2.58), its associated probability is less than 0.01.

### Preference for Internet-Based Cognitive Behavioral Therapy for Insomnia

All respondents were asked whether they had undergone CBT before drug therapy, and 47.7% (286/600) responded they had undergone CBT. There were no significant differences in the rate among the three groups: with insomnia using medication (51.5%,103/200), with insomnia not using medication (47.5%, 95/200), and without insomnia (44.0%, 88/200) (Table 5).

Notably, 57.2% (343/600) of the total respondents preferred CBT over drug therapy for insomnia. Among those with insomnia who were using an insomnia medication, the statement “I choose CBT” was significantly less frequent comparing the three respondent groups’ answers, and “I choose drug therapy” was significantly more frequent comparing the three respondent groups’ answers. In both groups of respondents with insomnia who were not taking an insomnia medication and the respondents without insomnia, the statement “I choose CBT” was significantly more frequent comparing the three respondent groups’ answers, and “I choose drug therapy” was significantly less frequent comparing the three respondent groups’ answers (Table 5). Although respondents with insomnia who were using insomnia medications had a relatively lower preference for CBT (40.5%, 81/200), both those with insomnia who were not taking an insomnia medication and the respondents without insomnia had a relatively higher preference for CBT (64.0%, 128/200 and 67.0%, 134/200, respectively).

**Table 5:** Preference for cognitive behavioral therapy (CBT) before drug therapy versus drug therapy, and preference for internet-based CBT versus face-to-face CBT.

Questions and answers		With insomnia, using medication (n=200)		With insomnia, not using medication (n=200)		Without insomnia, (n=200)		Total (N=600)	
		n (%)	Adjusted residual	n (%)	Adjusted residual	n (%)	Adjusted residual	n (%)	*P* value^a^
**Let’s assume that you received your diagnosis of insomnia from your doctor. Imagine that you were advised to participate in cognitive behavioral therapy (CBT) before drug therapy. In such a case, do you participate in CBT?**									
	Yes, I receive CBT	103 (51.5)	1.3	95 (47.5)	−0.1	88 (44.0)	−1.3	286 (47.7)	.32
	No. I do not receive CBT	97 (48.5)	−1.3	105 (52.5)	0.1	112 (56.0)	1.3	314 (52.3)	
**If you had to choose only one of the two as a treatment for insomnia, would you choose CBT or drug therapy?**									
	I choose CBT	81 (40.5)	−5.8^b,c^	128 (64.0)	2.4^b,d^	134 (67.0)	3.4^b,c^	343 (57.2)	<.001
	I choose drug therapy	119 (59.5)	5.8^b,c^	72 (36.0)	−2.4^b, d^	66 (33.0)	−3.4^b,c^	257 (42.8)	
**Other than CBT for insomnia in face-to-face sessions with a therapist, if there is a way to receive CBT via the internet and support from a therapist by email, which do you choose?**									
	I choose face-to-face CBT	119 (59.5)	2.3^b,d^	89 (44.5)	−2.9^b,c^	110 (53.0)	0.7	318 (53.0)	.009
	I choose CBT on the internet	81 (40.5)	−2.3^b,d^	111 (55.5)	2.9^b,c^	90 (47.0)	−0.7	282 (47.0)	

^a^From chi-square values.

^b^Cells with significant adjusted standardized residuals.

^c^The adjusted standardized residual is 2.58 or greater (or, alternatively, less than −2.58), its associated probability is less than 0.01.

^d^The adjusted standardized residual is 1.96 or greater (or, alternatively, less than −1.96), its associated probability is less than 0.05.

Of the total number of respondents, 47.0% (282/600) preferred internet-based CBT for insomnia to face-to-face CBT. In the group with insomnia taking an insomnia medication, the statement “I choose face-to-face CBT” was chosen significantly more frequently comparing the three respondent groups’ answers, and “I choose computerized CBT on the internet” was chosen less frequently when comparing the three respondent groups’ answers. Among the respondents with insomnia not using insomnia medication, “I choose face-to-face CBT” was chosen significantly less frequently when comparing the three respondent groups’ answers, and “I choose computerized CBT on the internet” was more chosen more frequently when comparing the three respondent groups’ answers (Table 5).

Although the group with insomnia using an insomnia medication had a relatively lower preference for internet-based CBT (40.5%, 81/200), the group with insomnia not using medication for it had a relatively higher preference for internet-based CBT (55.5%, 111/200).

## Discussion

### Principal Findings

Our Web-based survey of 600 individuals in Japan revealed that approximately half (57.2%, 343/600) of the respondents preferred CBT for insomnia to drug therapy, and half (47.0%, 282/600) preferred internet CBT for insomnia over face-to-face CBT.

Culver et al [21] reported that 57.7% of female veterans (N=1538) in the United States rated nonmedication treatment of insomnia as very acceptable, whereas only 33.5% rated medication treatment as very acceptable. Sedov et al [22] reported that 50.9% of a series of pregnant women in Canada (N=187) described CBT as their first choice for the treatment of insomnia, 11.8% selected pharmacotherapy, and 37.3% selected acupuncture if they experienced insomnia. Together, these results suggest a preference for CBT for insomnia over pharmacotherapy [22].

Regarding internet-based CBT, Cheung et al [23] conducted semistructured interviews in Australia, and they reported that 56.86% of their patients with insomnia (N=51) had a preference for face-to-face CBT, and 43.13% had a preference for internet-based CBT. Their results are similar to ours.

The treatment plan for an individual with insomnia should be tailored according to his or her values and preferences. Further research is needed to increase the availability of more effective internet CBT in addition to face-to-face CBT and pharmacotherapy.

### Limitations

Although our online survey obtained valuable information, our study has some limitations including the sampling methods. First, instead of random sampling, we used a stratified sample based on gender and age ranging from people in their twenties to their sixties from an internet inquiry to conduct our online survey. Individuals younger than 20 or older than 70 were excluded. However, all age groups are affected by insomnia, and the incidence tends to increase with age. In a study conducted in the United States, Ancoli-Israel et al [24] reported that 9% of 1000 subjects aged 18 years and older and 20% of 1000 subjects aged 65 years and older had chronic insomnia. Future studies should include people older than 70 years old. Older people have less access to online surveys than the general population. In that case, the use of a face-to-face survey and/or a telephone survey may be necessary to obtain data from people in their seventies. Second, we were unable to elucidate the insomnia severity or age. Third, the number of people using CBT was unclear. The questionnaires should include a number of insomnia patients using CBT with and without medication. Finally, the reason for preference was unclear. The questionnaires should include a reason for choice of medication, face-to-face CBT, and internet-based CBT.

### Conclusions

The responses to our online survey indicate that approximately half of the respondents preferred CBT over drug therapy for insomnia, and half preferred internet-based CBT for insomnia over face-to-face CBT.

## Abbreviations

CBT: cognitive behavioral therapy
